# Rheumatism Movement Cure

**Published:** 1914-03

**Authors:** Robert J. Burdette


### Rheumatism Movement Cure.

One day, not a great while ago, Mr. Middlerib read in his favorite paper a paragraph stating that the sting of a bee was a sure cure for rheumatism, and citing several remarkable instances in which people had been perfectly cured by this abrupt remedy. Mr. Middlerib thought of the rheumatic twinges that grappled his knees once in a while and made his life a burden.

He read the article several times and pondered over it. He understood that the stinging must be done scientifically and thoroughly. The bee, as he understood the article, was to be griped by the ears and set down upon the rheumatic joint and held there until it stung itself stingless. He had some misgivings about the matter. He knew it would hurt. He hardly thought it could hurt any worse than the rheumatism, and it had been so many years since he was stung by a bee that he had almost forgotten what it felt like. He had, however, a general feeling that it would hurt some. But desperate diseases require desperate remedies, and Mr. Middlerib was willing to undergo any amount of suffering if it would cure his rheumatism.

He contracted with Master Middlerib for a limited supply of bees; humming and buzzing about in the summer air, Mr. Middlerib did not know how to get them. He felt, however, that he could safely depend upon the instincts and methods of boyhood. He knew that if there was any way in heaven whereby the shyest bee that ever lifted a two-hundred-pound man off the clover could be induced to enter a wide-mouthed glass bottle, his son knew that way.

For the small sum of one dime Master Middlerib agreed to procure several, towit: six bees, sex and age not specified; but, as Mr. Middlerib was left in uncertainty as to the race, it was made obligatory upon the contractor to have three of them honey and three humble, or, in the generally accepted vernacular, bumblebees. Mr. M. did not tell his son what he wanted those bees for, and the boy went off on his mission with his head so full of astonishment that it fairly whirled. Evening brings all home, and the last rays of the declining sun fell upon Master Middlerib with a short, wide-mouthed bottle comfortably populated with hot, ill-natured bees, and Mr. Middlerib and a dime. The dime and the bottle changed hands. Mr. Middlerib put the bottle in his coat pocket and went into the house, eyeing everybody he met very suspiciously, as though he had made up his mind to sting to death the first person who said "bee" to him. He confided his guilty secret to none of his family. He hid his bees in his bedroom, and as he looked at them just before putting them away he half wished the experiment was safely over. He wished the imprisoned bees did not look so hot and cross. With exquisite care he submerged the bottle in a basin of water and let a few drops in on the heated inmates to cool them off.

At the tea table he had a great fright. Miss Middlerib, in the artless simplicity of her romantic nature, said:

"I smell bees. How the odor bring up——"

But her father glared at her and said, with superfluous harshness and execrable grammar: "Hush up! You don't smell nothing."

Whereupon Mrs. Middlerib asked him if he had eaten anything that disagreed with him, and Miss Middlerib said:

"Why, pa!" and Master Middlerib smiled as he wondered.

Bedtime at last, and the night was warm and sultry. Under various false pretenses, Mr. Middlerib strolled about the house until everybody else was in bed, and then he sough his room. He turned the lamp down until its feeble ray shone dimly as a death-light.

Mr. Middlerib disrobed slowly—very slowly. When at last he was ready to go lumbering into his peaceful couch, he heaved a profound sigh, so full of apprehension and grief that Mr. Middlerib, who was awakened by it, said if it gave him so much pain to come to bed perhaps he had better sit up all night. Mr. Middlerib choked another sigh, but said nothing and crept into bed. After lying still a few moments he reached out and got his bottle of bees.

It was not an easy thing to do to pick one bee out of the bottleful with his fingers and not get into trouble. The first bee Mr. Middlerib got was a little brown honey-bee, that wouldn't weigh half an ounce if you picked him up by the ears, but if you lifted him by the hind leg would weigh as much as the last end of a bay mule. Mr. Middlerib could not repress a groan.

"What's the matter with you?" sleepily asked his wife.

It was very hard for Mr. Middlerib to say he only felt hot, but he did it. He didn't have to lie about it, either. He did feel very hot indeed—about eighty-six all over, and one hundred and ninety-seven on the end of his thumb. He reversed the bee and pressed the warlike terminus of it firmly against the rheumatic knee.

It didn't hurt so badly as he thought it would.

It didn't hurt at all.

Then Mr. Middlerib remembered that when the honey-bee stabs a human foe it generally leaves its harpoon in the wound, and the invalid knew that the only thing this bee had to sting with was doing its work at the end of his thumb.

He reached his arm out from under the sheets and dropped this disabled atom of rheumatism liniment on the carpet. Then, after a second of blank wonder, he bgean to feel around for the bottle, and wished he knew what he did with it.

In the meantime strange things has been going on. When he caught hold of the first bee, Mr. Middlerib, for reasons, drew it out in such haste that for a time he forgot all the bottle and its remedial contents, and left it lying uncorked in the bed, between himself and his innocent wife. In the darkness there had been a quiet but general emigration from that bottle. The bees, their wings clogged with the water Mr. Middlerib had poured upon them to cool and tranquilize them, were crawling aimlessly about over the sheet. While Mr. Middlerib was feeling around for it, his ears were suddenly thrilled and his heart frozen by a wild, piercing scream from his wife.

"Murder!" she screamed. "Murder! Oh! Help me! Help! Help!"

Mr. Middlerib sat bolt upright in bed. His hair stood on end. The night was warm, but he turned to ice in a minute.

"Where in thunder," he said, with pallid lips, as he felt all over the bed in frenzied haste, "where in thunder are them infernal bees?"

And a large "bumble," with a sting as pitiless as the finger of scorn, just them climbed up the inside of Mr. Middlerib's nightshirt, until it got squarely between his shoulders, and then it felt for his marrow, and he said calmly:

"Here is one of them."

And Mrs. Middlerib felt ashamed of her feeble screams when Mr. Middlerib threw up both arms and, with a howl that made the windows rattle, roared:

"Take him off! Oh, land of Scott, somebody take him off!"

And when a little honey-bee began tickling the sole of Mrs. Middlerib's foot, she so shrieked that the house was bewitched, and immediately went into spasms.

The household was aroused by this time. Miss Middlerib and Master Middlerib and the servants were pouring into the room, adding to the general confusion by howling at random and asking irrelevant questions, while they gazed at the figure of a man a little on in years, arrayed in a long nightshirt, pawing fiercely at the unattainable spot in the middle of his back, while he danced an unnatural, weird, wicked-looking jig by the dim, religious light of the night-lamp. And while he danced and howled, and while they gazed and shouted, a navy-blue wasp, that Master Middlerib had put in the bottle for good measure and variety, and to keep the menagerie stirred up, had dried his legs and wings with a corner of the sheet, and after a preliminary circle or two around the bed to get up his motion and settle down to a working gait, he fired himself across the room, and to his dying day Mr. Middlerib will always believe that one of the servants mistook him for a burglar and shot him.

No one, not even Mr. Middlerib himself, could doubt that he was, at least for the time, most thoroughly cured of rheumatism. His own boy could not have carried himself more lightly or with greater agility. But the cure was not permanent, and Mr. Middlerib does not like to talk about it.—*Robert J. Burdette.*

